# Modern Medicine—Its Theory and Practice

**Published:** 1908-09

**Authors:** 


### Books and Magazines.

MODERN MEDICINE—ITS THEORY AND PRACTICE.—In original Contributions by American and Foreign Authors. Edited by William Osler, M. D., Regius Professor of Medicine in Oxford University, England; formerly Professor of Medicine in Johns Hopkins University, Baltimore; in the University of Pennsylvania, Philadelphia and in McGill University, Montreal. Assisted by Thomas McCrea, M. D., Associate Professor of Medicine and Clinical Therapeutics in Johns Hopkins University, Baltimore. In seven octavo volumes of about 900 pages each, illustrated. Volume IV, just ready. Price per volume: Cloth, $6.00, net; leather, $7.00, net; half morocco, $7.50, net. Lea & Febiger, Publishers, Philadelphia and New York. 1908.

In a work covering the vast domain of Internal Medicine it is no small merit to have the scheme logical and the division into volumes so arranged that the whole of a natural group can be taken from the shelf between a single pair of covers. It is a token of skill to do difficult things with apparent ease, and Professor Osler has certainly so managed the classification and division of subjects in Modern Medicine, two very important practical considerations.

The fourth volume, just from press, accordingly comprises all Diseases of the Circulatory System and of the Blood including the Spleen, Thymus and Lymph-Glands. Its list of authors exhibits the same editorial purpose and ability to know and to secure the best writer for each subject. As the plan for the whole work was of course developed before any part was undertaken, the seven volumes when assembled will constitute an even and complete library on General Medicine, and it may be remarked that as the leading authority on each subject was chosen without regard to nationality or geography, Modern Medicine, therefore, reflects the best human knowledge at the present time.

Good things sell themselves, and, conversely, a thing which sells itself is good. Modern Medicine answers this test by exhibiting a sale equal to five ordinary editions before it is even half issued. It is a practical consultant for every physician. In its pages he can post up on the methods and treatment developed by the most successful men the world over. Against such knowledge a man practicing on past ideas or individual experience is handicapped. To have the best equipment is compulsory in the long run, and the man who most quickly recognizes such aids as Modern Medicine gains both knowledge and time. Judging by its sale so far and its rate of progression Modern Medicine is destined to go into the library of every alert practitioner in America.

